# Synthesis and Antimicrobial Activity of Some New Thieno[2,3-*b*]thiophene Derivatives

**DOI:** 10.3390/molecules18044669

**Published:** 2013-04-19

**Authors:** Yahia Nasser Mabkhot, Nabila Abdelshafy Kheder, Ahmad M. Farag

**Affiliations:** 1 Department of Chemistry, Faculty of Science, King Saud University, P.O. Box 2455, Riyadh 11451, Saudi Arabia; 2 Department of Chemistry, Faculty of Science, Cairo University, Giza 12613, Egypt

**Keywords:** thieno[2,3-*b*]thiophene, nucleophilic addition, hydrazonoyl halides, *bis*-pyrazole, antimicrobial activity

## Abstract

A series of thieno[2,3-*b*]thiophene moiety-containing *bis*-cyanopyrazoles **5a**–**c**, *bis*-aminopyrazole **9** and *bis*-pyridazine derivatives **11** were synthesized and evaluated *in vitro* for their antimicrobial potential. The antimicrobial activity of some selected products was evaluated and showed good results. 5,5'-(3,4-Dimethylthieno[2,3-*b*]thiophene-2,5-diyl)bis(3-acetyl-1-(4-chlorophenyl)-1*H*-pyrazole-4-carbonitrile) (**5d**) was found to be more potent than the standard drug amphotericin B against *Geotricum candidum* and equipotent to amphotericin Bagainst *Syncephalastrum racemosum*. In addition, it was found to be equipotent to the standard drug Penicillin G against *Staphylococcus aureus*. Moreover, it was more potent than the standard drug streptomycin against *Pseudomonas aeruginosa* and *Escherichia coli*.

## 1. Introduction

Thieno[2,3-*b*]thiophenes possess important biological activities, including antiinflammatory [1,2], antimicrobial [3], analgesic [4] properties, antiproliferative activity [5], antagonism of α1 adrenoceptors [6] and prevention of cartilage destruction in articular diseases [7], In addition, thieno[2,3-*b*]thiophenes have shown useful reactivity as co-polymerization agents [8] and as semiconductors [9]. On the other hand, the synthesis of pyrazoles remains of great interest owing to their wide applications in the agrochemical and pharmaceutical industry due to their herbicidal, fungicidal, insecticidal, analgesic, antipyretic and anti-inflammatory properties [10,11]. Pyridazine compounds are also commonly used as anticancer [12], antituberculosis [13], antihypertensive [14], antifungal [15,16], or antimicrobial [17,18,19,20] agents, due to their intense biological activity. Encouraged by all these findings and in continuation of our ongoing research program investigating the utilisation of 3,3'-(3,4-dimethylthieno[2,3-*b*]thiophene-2,5-diyl)bis(3-oxopropanenitrile) (**1**) as a versatile and useful building block for the synthesis of a wide variety of *bis*-heterocyclic systems [21,22,23,24,25], we report in the present work an efficient and rapid method for the synthesis of a series of *bis*-cyanopyrazole, *bis*-aminopyrazole and *bis*-pyridazine derivatives containing thieno[2,3-*b*]thiophene as a base unit.

## 2. Results and Discussion

### 2.1. Chemistry

**Scheme 1:**
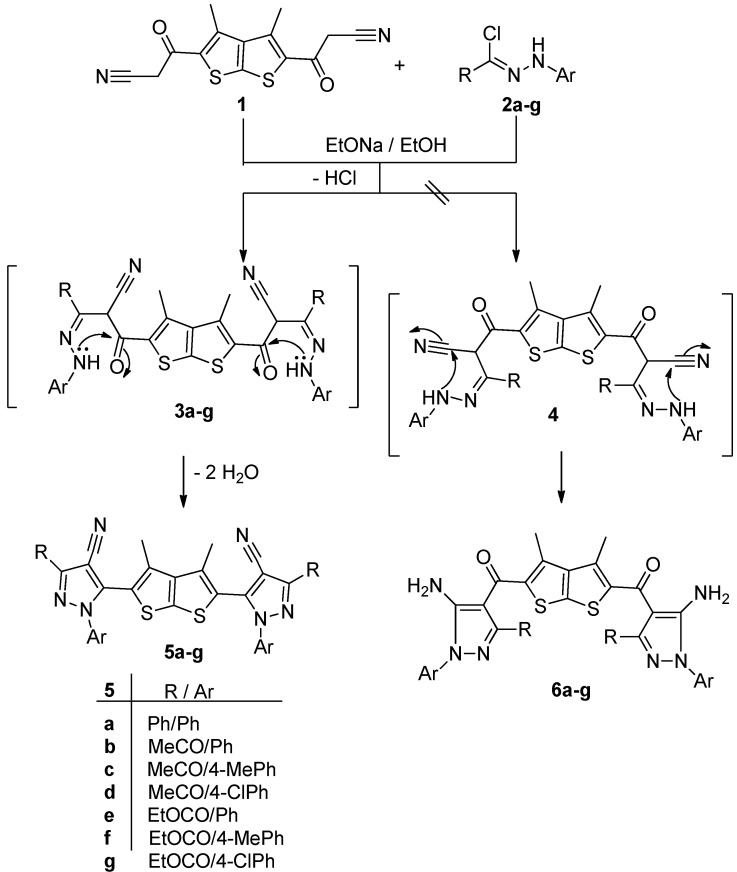
Synthesis of *bis*-cyanopyrazole derivatives **5a**–**g**.

Treatment of 3,3'-(3,4-dimethylthieno[2,3-*b*]thiophene-2,5-diyl)bis(3-oxopropanenitrile) (**1**) [21] with hydrazonoyl chloride **2a** [26] in ethanolic sodium ethoxide solution at room temperature furnished a single product, for which the two possible structures **5a** and **6a** can be envisaged (Scheme 1), but elemental analyses and spectral data were in complete accordance with the *bis-*cyanopyrazole structure **5a**. The IR spectrum of compound **5a** revealed the absence of amino and carbonyl bands. Its ^1^H-NMR spectrum revealed a singlet signal at δ 2.3 corresponding to methyl protons, in addition to aromatic multiplet protons in the δ 7.46–8.0 region. Moreover, the mass spectrum of product **5a** exhibited a molecular ion peak at *m/z* 654. Prompted by the foregoing results and to generalize this finding we also studied the reaction of the thieno[2,3-*b*]thiophene **1** with other hydrazonoyl chlorides **2b** [27] or **2c**,**d** [28] or **2e**–**g** [29] under the same experimental conditions and thus obtained the respective *bis-*cyanopyrazole derivatives **5b**,**c**. The structure of the isolated products **5b**–**g** were established from their elemental analyses and spectral data (see Experimental).

**Scheme 2:**
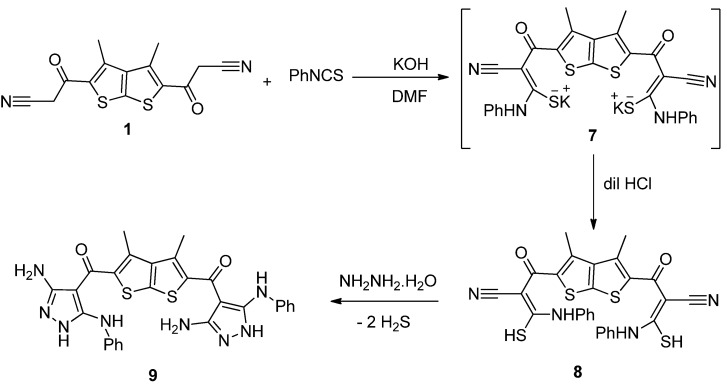
Synthesis of thieno[2,3-*b*]thiophene **8** and *bis*-aminopyrazole **9**.

Treatment of the thieno[2,3-*b*]thiophene **1** with phenyl isothiocyanate, in dimethylformamide, and in the presence of potassium hydroxide, at room temperature, followed by treatment with dilute hydrochloric acid, afforded a yellow-colored product identified 2-cyano-3-mercapto-3-(phenylamino)acryloyl)-3,4-dimethylthieno[2,3-*b*]thiophene-2-carbonyl)-3-mercapto-3-(phenylamino) acrylonitrile (**8**) (Scheme 2).

The structure of the latter product was confirmed on the basis of its elemental analysis and spectral data. The IR spectrum of compound **8** showed absorption bands at 3411, 2207 and 1714 cm^−1^ corresponding to NH, nitrile and carbonyl groups, respectively. Its ^1^H-NMR spectrum revealed a singlet signal at δ 2.49 and two D_2_O-exchangeable signals at δ 7.07 and 14.07 due to methyl, NH, and SH protons, respectively, in addition to aromatic multiplet protons in the δ 7.29–8.02 region. Refluxing of the thieno[2,3-*b*]thiophene **8** with hydrazine hydrate afforded *bis*-aminopyrazole **9**.

The ^1^H-NMR spectrum of **9** revealed the absence of SH proton and showed a singlet signal at δ 2.49 and two D_2_O-exchangeable signals at δ 4.55 and at δ 7.93 corresponding to methyl, NH_2_, and NH proton, respectively, in addition to aromatic multiplet protons in the region δ 7.31–7.45.

**Scheme 3:**
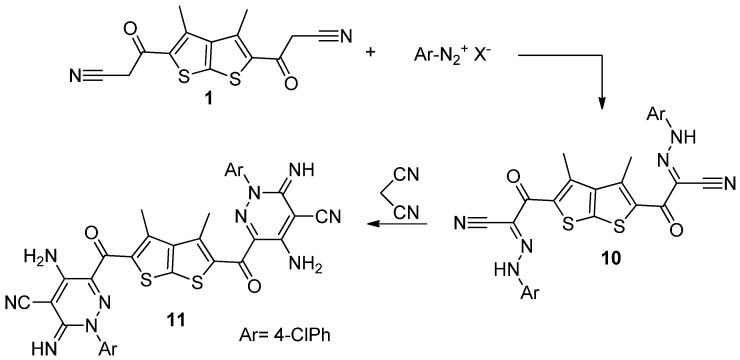
Synthesis of *bis*-pyridazine **11**.

Treatment of hydrazone **10** [21] with malononitrile in DMF, afforded *bis*-pyridazine **11** (Scheme 3). The structure of the latter product was established on the basis of its elemental analysis and spectral data. For example, its IR spectrum revealed two absorption band at v = 1651, 2214 cm^−1^, assignable to carbonyl and nitrile groups, in addition to bands at v = 3175–3410 cm^−1^ due to NH_2_ and NH. Its ^1^H-NMR spectrum showed two singlet signals at δ 2.49 ppm due to methyl groups, and two signals (D_2_O-exchangeable) at δ 4.1 and 7.48 ppm assignable to NH_2_, and NH protons, respectively. Moreover, the mass spectrum of the product **11** exhibited a molecular ion peak at *m/z* 711. 

### 2.2. Antimicrobial Evaluation

**Figure 1:**
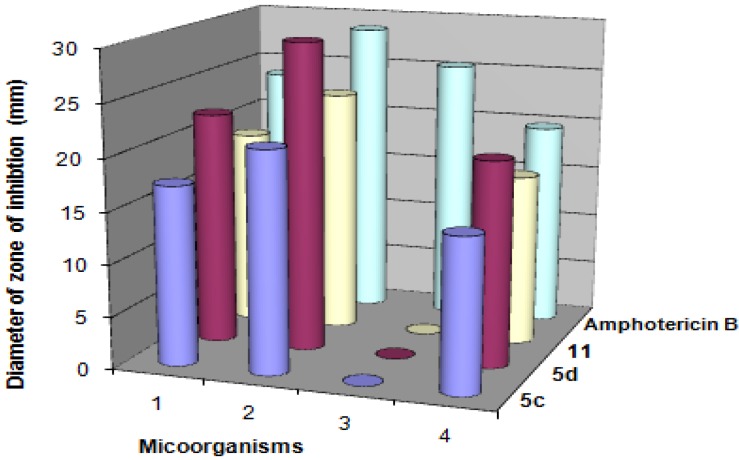
Antifungal activities of the synthesized compounds **5c**,**d** and **11** against micoorganisms—1. *Aspergillus fumgatus*; 2. *Geotrichum candidum*; 3. *Candida albicans*; 4. *Syncephalastrum racemosum*.

**Figure 2:**
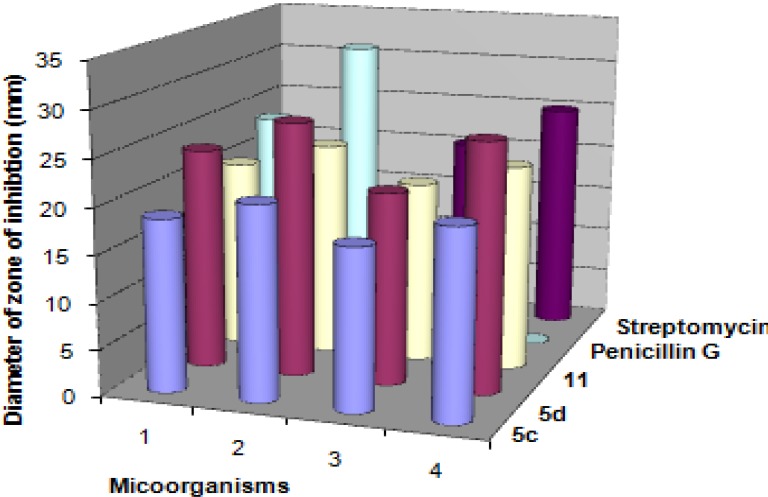
Antibacterial activities of the synthesized compounds **5c**,**d** and **11** against micoorganisms—1. *Staphylococcus aureus*; 2. *Bacillis subtilis*; 3. *Pseudomonas aeruginosa*; 4. *Escherichia coli*.

The newly synthesized target compounds **5b**,**c** and **11** were evaluated for their *in vitro* antibacterial activity against *Streptococcus pneumoniae* and *Bacillis subtilis* as examples of Gram-positive bacteria and *Pseudomonas aeruginosa* and *Escherichia coli* as examples of Gram-negative bacteria. They were also evaluated for their *in vitro* antifungal potential against the fungal strains *Aspergillus fumigatus*, *Syncephalastrum racemosum*, *Geotricum candidum* and *Candida albicans*. The organisms were tested against the activity of solutions of concentrations (5 μg/mL) and using inhibition zone diameter (IZD) in mm as criterion for the antimicrobial activity (agar diffusion method). The fungicide amphotericin B and the bactericides penicillin G and streptomycin were used as references to evaluate the potency of the tested compounds under the same conditions. The results are summarized in Table 1 and Table 2, Figure 1 and Figure 2.

**Table 1:** Antifungal activities of the synthesized compounds **5c**,**d** and **11**.

**Compound Tested**	**Micoorganisms**			
	***Aspergillus*** ***Fumigatus***	***Geotrichum*** ***Candidum***	***Candida*** ***albicans***	***Syncephalastrum*** ***racemosum***
**5c**	17.3 ± 0.4	21.3 ± 0.4	NA	14.8 ± 0
**5d**	22.4 ± 0.5	29.7 ± 0.2	NA	19.8 ± 0.8
**11**	18.9 ± 0.3	23.4 ± 0.4	NA	16.5 ± 0.2
***Amphotericin B***	23.7 ± 0.1	28.7 ± 0.2	25.4 ± 0.1	19.7 ± 0.2

NA: No activity, data are expressed in the form of mean ± SD.

**Table 2:** Antibacterial activities of the synthesized compounds **5c**,**d** and **11**.

**Compound Tested**	**Gram-Positive Bacteria**		**Gram-Negative Bacteria**	
	***S. aureus***	***B. subtilis***	***P. aeruginosa***	***E. coli***
**5c**	18.6 ± 0.3	20.9 ± 0.5	17.4 ± 0.2	20.3 ± 0.4
**5d**	23.8 ± 0.5	27.4 ± 0.6	20.7 ± 0.2	26.8 ± 0.2
**11**	20.4 ± 0.4	23.1 ± 0.2	19.6 ± 0.1	22.2 ± 0.5
***Penicillin G***	23.8 ± 0.2	32.4 ± 0.3	-	-
***Streptomycin***	-	-	20.3 ± 0.1	24.9 ± 0.3

NA: No activity, data are expressed in the form of mean ± SD. Mean zone of inhibition in mm ± Standard deviation beyond well diameter (6 mm) produced on a range of environmental and clinically pathogenic microorganisms using (5 mg/mL) concentration of tested samples.

The results in Table 1 revealed that 5,5'-(3,4-dimethylthieno[2,3-*b*]thiophene-2,5-diyl)bis(3-acetyl-1-(4-chlorophenyl)-1*H*-pyrazole-4-carbonitrile) (**5d**) was found to be more potent than the standard drug Amphotericin B against *Geotricum candidum*. Also, it was equipotent to the standard drug, amphotericin B against *Syncephalastrum racemosum*. In addition, it was found to be equipotent to the standard drug penicillin G against *Staphylococcus aureus*. Moreover, it was more potent than the standard drug streptomycin against *Pseudomonas aeruginosa* and *Escherichia coli*. All compounds exhibited almost no activity against *Candida albicans.* The structure antimicrobial activity relationship of the synthesized compounds **5c** and **5d** revealed that the maximum activity was attained with compound **5d**, having a pyrazole nucleus with chloro substituent in the phenyl group.

## 3. Experimental

All melting points were measured on a Gallenkamp melting point apparatus (Weiss-Gallenkamp, London, UK). The infrared spectra were recorded in potassium bromide disks on a pye Unicam SP 3300 and Shimadzu FT IR 8101 PC infrared spectrophotometers (Pye Unicam Ltd. Cambridge, England and Shimadzu, Tokyo, Japan, respectively). The NMR spectra were recorded on a Bruker VX-500 NMR spectrometer (Varian, Palo Alto, CA, USA). ^1^H-NMR spectra were run at 500 MHz and ^13^C-NMR spectra were run at 125 MHz in deuterated dimethylsulphoxide (DMSO-*d_6_*). Chemical shifts were related to that of the solvent. Mass spectra were recorded on a Shimadzu GCMS-QP 1000 EX mass spectrometer at 70 e.V. Elemental analyses were carried out at the Micro-analytical Center of Cairo University, Giza, Egypt. The biological evaluation of the products **5c**,**d** and **11** were carried out in the Medical Mycology Laboratory of the Regional Center for Mycology and Biotechnology of Al-Azhar University, Cairo, Egypt. Thieno[2,3-*b*]thiophene **1** [21], hydrazonoyl chlorides **2a** [26], **2b** [27], **2c**,**d** [28], **2e**–**g** [29], and hydrazone **10** [21] were prepared following the literature procedures.

### 3.1. Reaction of Thieno[2,3-*b*]thiophene **1** with Hydrazonoyl Halides

#### General Procedure

Thieno[2,3-*b*]thiophene **1** (0.3 g, 1 mmol) was added to an ethanolic sodium ethoxide solution [prepared from sodium metal (46 mg, 2 mmol) and absolute ethanol (20 mL)] with stirring. After stirring the resulting solution for 15 min, the appropriate hydrazonoyl halide **2a**–**g** (2 mmol) was added portionwise and the reaction mixture was stirred further for 12 h at room temperature. The solid that formed was filtered off, washed with water and dried. Recrystallization from the proper solvent afforded the corresponding pyrazole derivatives **5a**–**g** in 45–55% yields.

*5,5'-(3,4-Dimethylthieno*[2,3-*b*]*thiophene-2,5-diyl)bis(1,3-diphenyl-1H-pyrazole-4-carbonitrile)* (**5a**). Yield 48%, mp > 300 °C (DMF/EtOH); IR (KBr) v max: 2226 (C≡N), 2909 (aliphatic CH) cm^−1^; ^1^H-NMR: δ 2.3 (s, 6H, 2CH_3_), 7.46–8.0 (m, 20H, ArH); ^13^C-NMR: δ 13.88, 93.26, 114.24, 115.42, 122.91, 125.08, 126.46, 129.21, 129.49, 129.92, 133.65, 138.08, 140.27, 142.54, 144.54, 146.09, 152.23. MS *m/z* (%) 657 (11.64), 656 (30.71), 655 (M^+^, 60.86), 654 (63.43), 637 (5.22), 620 (5.82), 619 (6.42), 166 (26.18), 77 (87.63). Anal. Calcd for C_40_H_26_N_6_S_2_ (654.80): C, 73.37; H, 4.00; N, 12.83; S, 9.79. Found: C, 73.28; H, 4.10; N, 12.77; S, 9.67%.

*5,5'-(3,4-Dimethylthieno*[2,3-*b*]*thiophene-2,5-diyl)bis(3-acetyl-1-phenyl-1H-pyrazole-4-carbonitrile)* (**5b**). Yield 45%, mp > 300 °C (DMF/EtOH); IR (KBr) v max: 1680 (C=O), 2226 (C≡N), 2915 (aliphatic CH) cm^−1^; ^1^H-NMR: δ 2.16 (s, 6H, 2CH_3_), 2.30 (s, 6H, 2CH_3_), 7.46–7.98 (m, 10H, ArH); MS *m/z* (%) 587 (M^+^, 12.82), 585 (82.77), 464 (33.57), 166 (26.18), 77 (87.63). Anal. Calcd for C_32_H_22_N_6_O_2_S_2_ (586.69): C, 65.51; H, 3.78; N, 14.32; S, 10.93. Found: C, 65.62; H, 3.85; N, 14.44 S, 11.03%.

*5,5'-(3,4-Dimethylthieno*[2,3-*b*]*thiophene-2,5-diyl)bis(3-acetyl-1-p-tolyl-1H-pyrazole-4-carbonitrile)* (**5c**). Yield 45%, mp > 300 °C (DMF/EtOH); IR (KBr) v max: 1680 (C=O), 2226 (C≡N), 2915 (aliphatic CH) cm^−1^; ^1^H-NMR: δ 2.16 (s, 6H, 2CH_3_), 2.30 (s, 6H, 2CH_3_), 2.79 (s, 6H, 2CH_3_); 7.46–7.98 (m, 8H, ArH); MS *m/z* (%) 615 (M^+^, 18.25), 192 (47.53) 166 (26.18). Anal. Calcd for C_34_H_26_N_6_O_2_S_2_ (614.74): C, 66.43; H, 4.26; N, 13.67; S, 10.43. Found: C, 66.52; H, 4.36; N, 13.58; S, 10.23%.

*5,5'-(3,4-Dimethylthieno*[2,3-*b*]*thiophene-2,5-diyl)bis(3-acetyl-1-(4-chlorophenyl)-1H-pyrazole-4-carbonitrile)* (**5d**). Yield 75%, mp > 300 °C (DMF/EtOH); IR (KBr) v max: 1680 (C=O), 2230 (C≡N), 2990 (aliphatic CH) cm^−1^; ^1^H-NMR: δ 2.16 (s, 6H, 2CH_3_), 2.30 (s, 6H, 2CH_3_), 7.46–7.98 (m, 8H, ArH); MS *m/z* (%) 655 (M^+^, 8.25), 192 (47.53) 166 (26.18). Anal. Calcd for C_32_H_20_Cl_2_N_6_O_2_S_2_ (655.58): C, 58.63; H, 3.07; N, 12.82; Cl, 10.82; S, 9.78. Found: C, 58.72; H, 3.16; N, 12.77; Cl, 10.67; S, 9.93%.

*Diethyl 5,5'-(3,4-dimethylthieno*[2,3-*b*]*thiophene-2,5-diyl)bis(4-cyano-1-phenyl-1H-pyrazole-3-carboxylate)* (**5e**). Yield 43%, mp > 300 °C (DMF/EtOH); IR (KBr) v max: 1680 (C=O), 2230 (C≡N), 2980 (aliphatic CH) cm^−1^; ^1^H-NMR: δ 1.16 (t, 6H, 2CH_3_), 2.30 (s, 6H, 2CH_3_), 4.23 (q, 4H, 2CH_2_), 7.46–7.98 (m, 10H, ArH); MS *m/z* (%) 647 (M^+^, 17.66), 648 (M^+^+1, 44.79), 192 (47.53) 166 (26.18), 77 (87.63). Anal. Calcd for C_34_H_26_N_6_O_4_S_2_ (646.74): C, 63.14; H, 4.05; N, 12.99; S, 9.92. Found: C, 63.25; H, 4.13; N, 12.83; S, 10.00%.

*Diethyl 5,5'-(3,4-dimethylthieno*[2,3-*b*]*thiophene-2,5-diyl)bis(4-cyano-1-p-tolyl-1H-pyrazole-3-carboxylate)* (**5f**). Yield 77%, mp > 300 °C (DMF/EtOH); IR (KBr) v max: 1680 (C=O), 2230 (C≡N), 3000 (aliphatic CH) cm^−1^; ^1^H-NMR: δ 1.16 (t, 6H, 2CH_3_), 2.30 (s, 6H, 2CH_3_), 2.78 (s, 6H, 2CH_3_), 4.23 (q, 4H, 2CH_2_), 7.46–7.98 (m, 8H, ArH); MS *m/z* (%) 676 (33.77), 675 (M^+^, 24.71), 192 (47.53) 166 (26.18). Anal. Calcd for C_36_H_30_N_6_O_4_S_2_ (674.79): C, 64.08; H, 4.48; N, 12.45; S, 9.50. Found: C, 64.18; H, 4.37; N, 12.38; S, 9.37%.

*Diethyl 5,5'-(3,4-dimethylthieno*[2,3-*b*]*thiophene-2,5-diyl)bis(1-(4-chlorophenyl)-4-cyano-1H-pyrazole-3-carboxylate)* (**5g**). Yield 56%, mp > 300 °C (DMF/EtOH); IR (KBr) v max: 1680 (C=O), 2230 (C≡N), 2980 (aliphatic CH) cm^−1^; ^1^H-NMR: δ 1.16 (t, 6H, 2CH_3_), 2.30 (s, 6H, 2CH_3_), 4.23 (q, 4H, 2CH_2_), 7.46–7.98 (m, 8H, ArH); MS *m/z* (%) 715 (M^+^, 16.95), 112 (18.34), 111 (49.94). Anal. Calcd for C_34_H_24_Cl_2_N_6_O_4_S_2_ (715.63): C, 57.06; H, 3.38; Cl, 9.91; N, 11.74; S, 8.96. Found: C, 57.18; H, 3.29; Cl, 9.91; N, 11.83; S, 9.85%.

*2-Cyano-3-mercapto-3-(phenylamino)acryloyl)-3,4-dimethylthieno*[2,3-*b*]*thiophene-2-carbonyl)-3-mercapto-3-(phenylamino)acrylonitrile* (**8**). To a stirred solution of potassium hydroxide (0.11 g, 2 mmol) in DMF (20 mL) was added thieno[2,3-*b*]thiophene **1** (0.3 g, 1 mmol). After stirring for 30 min, phenyl isothiocyanate (0.27 g, 0.24 mL, 2 mmol) was added to the resulting mixture and stirring was continued for 6 h, then poured onto curshed ice containing hydrochloric acid. The formed solid product was filtered off, washed with water, dried and finally recrystallized from dioxane/ EtOH mixture to afford compound **8** in 82% yield, mp 233–234 °C; IR (KBr) v 3411 (NH), 2207 (C≡N), 1714 (C=O) cm^−1^; ^1^H-NMR: δ 2.49 (s, 6H, 2CH_3_), 7.07 (s, 2H, D_2_O-exchangeable 2NH), 7.29–8.02 (m, 10H, ArH), 14.07 (s, 1H, D_2_O-exchangeable SH). MS *m/z* (%) (M^+^, not detected), 353 (7.19), 203 (6.36), 166 (6.51), 92 (15.64), 77 (71.78). Anal. Calcd for C_28_H_20_N_4_O_2_S_4_ (572.74): C, 58.72; H, 3.52; N, 9.78; S, 22.39. Found: C, 58.66; H, 3.61; N, 9.88; S, 22.24%.

*Synthesis of 3,4-dimethylthieno*[2,3-*b*]*thiophene-2,5-diyl)bis((3-amino-5-(phenylamino)-1H-pyrazol-4-yl)methanone)* (**9**). A mixture of compound **8** (0.572 g, 1 mmol) and hydrazine hydrate 98% (2 mmol) were heated on a steam bath for 1h, then left to cool. The reaction mixture was triturated with ethanol and the resulting solid product was filtered off and recrystalized from DMF/ethanol to give *bis*-pyrazole **9** in 48% yield, mp > 300 °C; IR (KBr) v 3400 (NH), 3465 (NH), 1714 (C=O) cm^−1^; ^1^H-NMR: δ 2.49 (s, 6H, 2CH_3_), 4.55 (s, 4H, D_2_O-exchangeable 2NH_2_), 7.26 (s, 2H, D_2_O-exchangeable 2NH), 7.93 (s, 2H, D_2_O-exchangeable 2NH), 7.31–7.45 (m, 10H, ArH). Anal. Calcd for C_28_H_24_N_8_O_2_S_2_ (568.67): C, 59.14; H, 4.25; N, 19.70; S, 11.28. Found: C, 59.22; H, 4.34; N, 19.61; S, 11.33%.

### 3.2. Reaction of Hydrazone **10** with Malononitrile

To an ethanolic solution of hydrazone **10** (0.58 g, 1 mmol) and malononitrile (0.132 g, 2 mmol) was added few drops of piperidine and the reaction mixture was refluxed for 4 h. The solid product was collected by filtration, washed with ethanol and purified by crystallisation from DMF to afford the *bis*-pyridazine **11** in 66% yield, mp 208–209 °C; IR (KBr) v 3175, 3244 (NH_2_), 3333 (NH) 2214 (C≡N), 1651 (C=O) cm^−1^; ^1^H-NMR (DMSO-*d_6_*): δ 2.49 (s, 6H, 2CH_3_), 4.1 (s, 4H, D_2_O-exchangeable 2NH_2_), 7.31–7.48 (m, 10H, ArH + 2NH); MS *m/z* (%) 711 (M^+^, 30.38), 710 (38.26), 272 (34.65), 244 (21.45), 111 (20.70) . Anal. Calcd for C_32_H_20_Cl_2_N_10_O_2_S_2_ (711.60): C, 54.01; H, 2.83; N, 19.68; Cl, 9.96; S, 9.01. Found: C, 54.15; H, 2.78; N, 19.59; Cl, 9.95; S, 8.98%.

## 4. Conclusions

A novel series of *bis*-cyanopyrazole, *bis*-aminopyrazole and *bis*-pyridazine derivatives with thieno[2,3-*b*]thiophene moieties were synthesized and evaluated for their antimicrobial activities. In general, most of the tested compounds was found to be equipotent or more potent than the standard drugs.
